# The fat and the bad: Mature adipocytes, key actors in tumor progression and resistance

**DOI:** 10.18632/oncotarget.18038

**Published:** 2017-05-20

**Authors:** Minh Ngoc Duong, Aline Geneste, Frederique Fallone, Xia Li, Charles Dumontet, Catherine Muller

**Affiliations:** ^1^ Department of Oncology/CHUV-UNIL, Biopole 3, Epalinges, Switzerland; ^2^ Centre de Recherche en Cancérologie de Lyon (CRCL), INSERM UMR 1052/CNRS 5286, Lyon, France; ^3^ Institut de Pharmacologie et de Biologie Structurale (IPBS), Université de Toulouse, CNRS, UPS, Toulouse, France; ^4^ Hospices Civils de Lyon, Lyon, France

**Keywords:** adipocytes, adipose tissue, cancer progression, invasion, resistance

## Abstract

Growing evidence has raised the important roles of adipocytes as an active player in the tumor microenvironment. In many tumors adipocytes are in close contact with cancer cells. They secrete various factors that can mediate local and systemic effects. The adipocyte-cancer cell crosstalk leads to phenotypical and functional changes of both cell types, which can further enhance tumor progression. Moreover, obesity, which is associated with an increase in adipose mass and an alteration of adipose tissue, has been established as a risk factor for cancer incidence and cancer-related mortality. In this review, we summarize the mechanisms of the adipocyte-cancer cell crosstalk in both obese and lean conditions as well as its impact on cancer cell growth, local invasion, metastatic spread and resistance to treatments. Better characterization of cancer-associated adipocytes and the key molecular events in the adipocyte-cancer cell crosstalk will provide insights into tumor biology and suggest efficient therapeutic opportunities.

## INTRODUCTION

Adipose tissue (AT) is one of the main components of the human body. Imaging methods, including computed tomography, magnetic resonance imaging and ultrasound, have allowed the quantification of adipose tissue. The latter was estimated to represent 18–25% of the body mass in a reference man, 25–31% in a reference woman, and higher percentage in overweight people [1]. Based on its biological functions, AT can be classified into two main types: white adipose tissue (WAT) localized subcutaneously, surrounding visceral organs or in the breast in females (mammary adipose tissue or MAT) [2] and brown adipose tissue (BAT) in paracervical and supraclavicular regions [3–5]. While WAT is specialized in storing energy and is an important endocrine organ involved mainly in the control of weight regulation, the BAT is the main tissue regulating thermogenesis in response to food intake and cold. A new type of adipocytes has been recently described, referred to as the brite, also called beige, adipocytes. Beige adipocytes share some common features with brown adipocytes, such as the expression of mitochondrial uncoupling protein 1 (UCP1) despite the fact that they reside in WAT, predominantly in subcutaneous WAT [6]. Finally, several « non typical » AT have been described such as the one included in the bone marrow. Bone marrow adipocytes (BM-Ad) possess unique profiles and have been proposed to exhibit features of beige or brown-like adipocytes [7–9].

WAT, which is the main focus of this review, is highly complex in terms of cellular composition, including mature adipocytes, pre-adipocytes, fibroblasts, pericytes, endothelial cells, and immune cells. Although adipocytes were estimated to account for only 14–24% of the adipose tissue cell populations [10, 11], due to their large size, adipocytes are still considered as the major component of AT. For a long time, adipocytes from WAT were viewed as a simple passive energy storage depot. However, since the discovery of leptin in 1994, the role of adipocytes has evolved into an active source of various paracrine and endocrine factors. These adipocyte-secreted factors constitute a group of molecules named adipokines, including common growth factors, hormones, cytokines, chemokines, and also specific factors such as leptin, adiponectin. To date, more than 400 factors have been reported to be released by adipocytes, and this number is still increasing [12]. These adipocyte-derived factors, acting locally or systemically, could play important roles in the growth, local invasion, metastatic spread and resistance to treatments of different types of cancer. Studying the role of adipocytes in cancer occurrence or progression is of major clinical interest due to the established link between obesity and cancer. Obesity is a pathological condition accompanied by an excessive fat deposition and is often estimated through the measure of body mass index (BMI) (weight in kg / height in m^2^). It is due to an imbalance between dietary energy intake and energy output [13]. The excessive energy intake in obese people is stored mainly in adipose tissue that increases in mass. The expansion of adipose tissue is essentially due to an increase in adipocyte volume (hypertrophy) and to a lesser extent to an increase in adipocyte cell number (hyperplasia) [14, 15]. Importantly, obesity is not only related to an increase in adipose quantity, but also an alteration of adipose quality. Obese adipose tissue has been characterized as being in a chronic inflammation state, with remodeling of local adipose tissue and dysregulation of secreted adipokines [16]. In addition to cardiovascular disease and type 2 diabetes mellitus, overweight and obesity are now established risk factors for cancer and cancer-related mortality. Excess body weight increases incidence of several types of cancer including, among the most frequent, oesophageal adenocarcinoma, colorectal, endometrial and post-menopausal breast cancer. Obesity also portends worse cancer-specific outcomes after diagnosis in several tumor types including those of the breast (independently of menopausal status), oesophagus, colon, prostate and others [17, 18]. This relationship has major consequences in public health since the prevalence of overweight and obesity has been increasing worldwide over the past decades and reaches alarming proportions. According to the World Health Organization (WHO), worldwide obesity has more than doubled since 1980 and in 2014, more than 1.9 billion adults were overweight and of these over 600 million were obese. It has been estimated that by 2025, global obesity prevalence will continue to increase, reaching 18% in men and more than 21% in women [19].

The precise mechanisms underlying the obesity–cancer link are not yet well understood. However, it is tempting to speculate that, within a context of obesity, adipocytes, due to their dysfunctional state, could be more prone to contribute to a favorable environment for the development of tumor cells. This review will summarize the impact of mature adipocytes on the biological characteristics of cancer cells. We will focus on adipocytes from WAT, referred as adipocytes in this review, since they are the most documented. However, the emerging role of BM-Ad on cancer will be also highlighted across the review. The crosstalk between adipocytes and cancer cells will be detailed in both lean and obese conditions, and its potential therapeutic implication for cancer treatment will be discussed. Due to space limitations, the role of other cellular components of AT in cancer progression will not be discussed. However, very interesting results have been obtained with adipose progenitors whose implication in tumor progression has been highlighted for example in a recent review on breast cancer [20]. Besides, the impact of obesity-related metabolic disorders on cancer, such as hyperinsulinemia and insulin resistance, has been reviewed elsewhere [17, 21]. Moreover, the modification of the immune environment (including the recruitment of pro-inflammatory macrophages) arising in AT during obesity is also probably very important in terms of cancer progression. Readers interested in these aspects could refer to recent reviews [22, 23].

### Adipocytes and cancer cells: close neighbours

Regarding the role of AT in cancer progression, local tissue-specific effects must be viewed with particular attention. Due to the distribution of AT in different organs, adipocytes are in close contact with cancer cells in many solid tumors during tumor growth, local invasion or bone metastasis as well as in hematological malignancies. The most prominent example of this proximity is in breast. Structurally, a normal breast is composed of an epithelial compartment (mammary gland) embedded in a stroma referred to as the mammary fat pad. Unlike murine mammary fat pad which is mainly composed of adipocytes, the human mammary fat pad is also enriched in connective tissue [24]. Therefore, while murine mammary epithelium is directly adjacent to adipocytes, human mammary epithelium is separated from adipocytes by a fibrous layer. However, in certain situations such as mammary involution or tumor invasion, the mammary extracellular matrix is remodeled, resulting in a direct contact between epithelial cells and adipocytes [25]. Similarly, the vicinity between adipocytes and cancer cells has also been observed in invasive melanoma, prostate, colon and ovarian cancers [26–28]. Furthermore, it is worth noting that the bone marrow is a niche for hematological malignancies and a metastatic site of many cancers, such as breast and prostate. Adipocytes constitute a major component of the bone marrow stroma [8, 9] and importantly, the adipose mass is increased with obesity and aging which could have an impact on cancer development in obese and/or elderly patients [29, 30].

**Figure 1:**
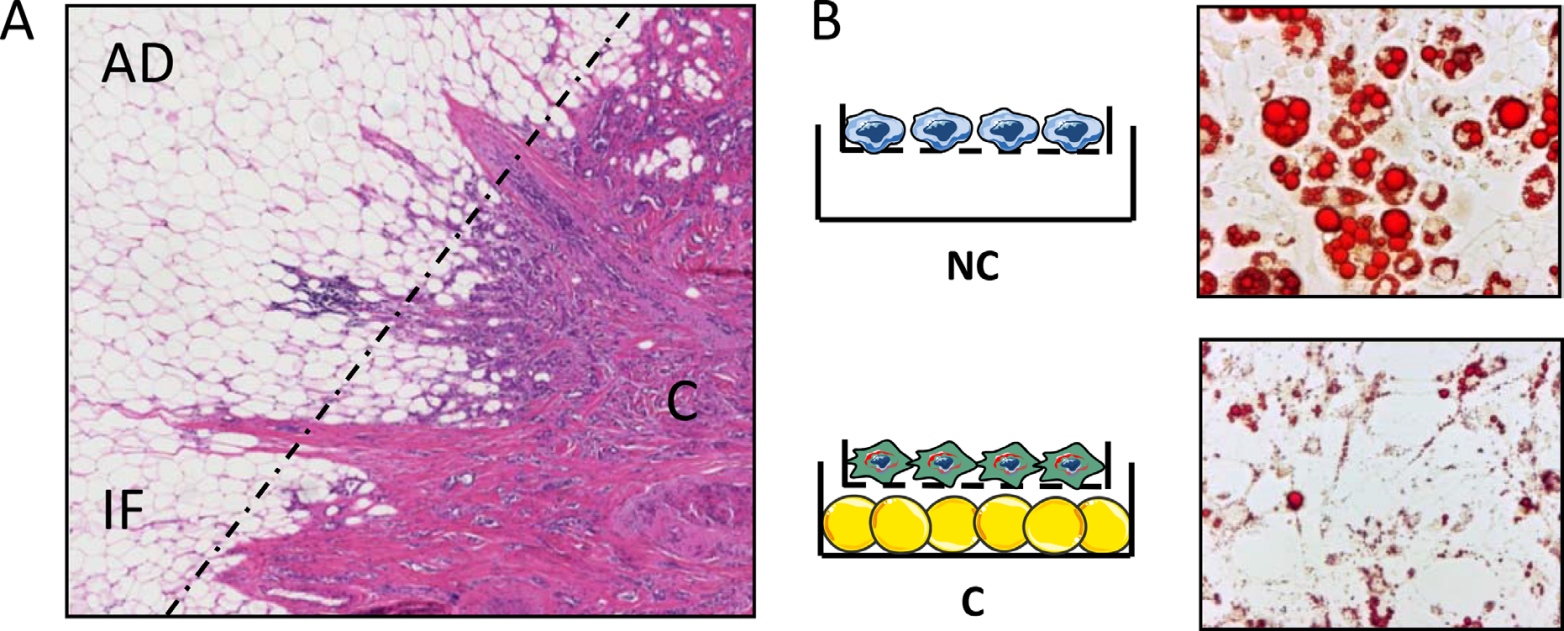
In breast cancers, adipocytes localized at the tumor invasive front undergo decrease in size and lipid content, a process that can be recapitulated *in vitro*, in co-culture assays (**A**) Histological examination of an invasive breast tumor after H&E staining (original magnification ×100). AD, adipose tissue; IF, invasive front (indicated by a dashed line); (**C**) tumor center. Note that at the invasive front, the size of adipocytes is reduced. (**B**) Mature adipocytes cocultivated in the presence (C) or absence (NC) of breast cancer cells were stained with oil Red O. The cocultivated adipocytes exhibit a decrease in the number and size of lipid droplets.

As a consequence of the close localization between adipocytes and invasive cancer cells, adipocytes in the vicinity of cancer cells display profound phenotypic and functional alterations. Histological images of solid tumors consistently showed a decrease in both cell number and cell size of adipocytes located at the invasive front compared to adipocytes distant from the tumor [28, 31]. Moreover, at the tumor center, there is an increase in the ratio of fibroblast-like cells [32], suggesting a “dedifferentiation” of adipocytes induced by cancer cells. We have shown that these phenotypical changes can be reproduced *in vitro* using a co-culture system where the two populations are separated by an insert. Co-culture of adipocytes with cancer cells for 3 to 5 days lead to adipocyte delipidation and decreased expression of adipocyte markers such as Ap2 (FABP4), adiponectin, and hormone-sensitive lipase [31]. Additionally, co-cultured adipocytes displayed modifications of their secretome, notably an upregulation of osteopontin, matrix metalloproteinase 11 and inflammatory cytokines such as TNFa, IL-6 and IL-1β [31, 33]. Such an activated phenotype has been confirmed *in vivo* at the invasive front of human breast tumors [31, 34] (Figure 1). Together, these *in vitro* and *in vivo* data indicate that adipocytes are modified by cancer cells to acquire characteristics different from those of naive adipocytes. We named them cancer-associated adipocytes (CAAs) [35]. Moreover, upon prolonged exposure to tumor cells, mature adipocytes completely lose their lipid content and exhibit fibroblast-like morphology highlighting that they contribute to the cancer-associated fibroblast population [36], which are known to further enhance tumor progression and metastasis [37]. In fact, in all tumors growing in an adipose tissue-dominated microenvironment (gastric, breast, colon, renal, prostate and ovarian cancers and melanoma), it is now admitted that when the tumor invades the surrounding AT, adipocytes disappear, fibroblast-like cells accumulate, and a desmoplastic stroma ensues (for review see [38]). Similar lipid loss has been observed *in vitro* when BM-Ad (obtained from *ex vivo* differentiation of bone marrow mesenchymal stem cells) are cocultured with prostate cancer cells [39], suggesting that CAAs might also occurs at bone metastatic sites. The origin of these phenotypic and functional alterations in adipocytes is only partially characterized. In breast cancer, we have demonstrated that occurrence of the CAA phenotype depends on the reactivation of the Wnt/b-catenin pathway in response to Wnt3a secreted by tumor cells [36]. Thus, this crosstalk should be taken in account when considering the paracrine role of AT, since consistent results show that adipocytes are not inert actors in regards to their surrounding within the tumor. Since most of the experimental studies performed emphasize the paracrine role of adipocytes, we will focus on this aspect of the adipocyte/cancer cell crosstalk. Nevertheless, AT also constitutes an active endocrine organ that can have far-reaching effects on the physiology of other tissues. To understand the endocrine effect of adipose tissue on cancer the reader is referred to reviews on that topic [40–42].

### Role of mature adipocytes in cancer progression: effect on tumor growth, local invasion and metastasis

### Adipocytes support tumor growth

Several studies have demonstrated the role of adipocytes and AT in the support and the promotion of tumor growth. Elliott *et al*. showed that murine mammary carcinoma cells grew better when injected in the mesenteric and ovarian fat pad or in the mammary gland than in subcutis or the peritoneal cavity. Moreover, co-transplantation of these cancer cells with mammary or ovarian fat fragments into the subcutis increased the tumor growth [43]. Manabe and colleagues showed that mature rat adipocytes, but not rat pre-adipocytes, increased tumor growth of estrogen receptor-positive (ER+) breast carcinoma cell lines [44]. More recently, Nieman *et al*. showed that co-culture of human adipocytes with ovarian cancer cells promoted cancer cell growth both *in vitro* and *in vivo* [28]. A similar effect of promoting tumor growth by adipocytes has been observed in prostate cancer, colon cancer and melanoma *in vitro* [45–48]. Emerging studies suggest that the growth promoting-effect of adipocytes is also observed at bone metastatic sites with an increase in bone tumor burden after intratibial injection of prostate cancer and melanoma cells in high fat diet-induced obese mice [39, 49]. Despite the evidence that adipocytes could promote tumor growth, the relationship between tumor growth and adipocytes might be more complex than that initially thought. For example, we have shown that some breast tumor cell lines co-cultivated with adipocytes exhibit increased proliferation, but this was not a general effect in contrast to the effect on invasion [31]. Similar results were obtained with prostate and melanoma cell lines ([50], Muller *et al*., unpublished results). Interestingly, a recent work performed *in vivo* in a large number of breast tumors (around 1000) showed that estrogen receptor-negative breast tumors at the close proximity of adipose tissue exhibited significantly lower mitotic index when compared with that of the tumor cells on the gland side [51]. In association to our *in vitro* results, these compelling results emphasize that the effect of adipocytes on tumor cell proliferation might be more complex than initially thought and depend on the tumor model used. This aspect will clearly need additional studies. Nevertheless, since a growth-promoting effect has been largely reported in several models, the mechanisms involved in this phenomenon will be described in the following paragraph.

### Adipocytes secrete adipokines promoting tumor growth

Many tumor cells express receptors for the adipokines secreted by adipocytes, which can affect tumor growth. These adipocyte-derived factors include mainly leptin, adiponectin, estrogen, insulin-like growth factor 1 (IGF-1) and hepatocyte growth factor (HGF).

### Leptin and adiponectin

The role of leptin in tumorigenesis was suggested by the high expression of the leptin receptor (ObR) in several cancer cells, such as breast, stomach, colon, ovarian cancers and leukemia [52–56]. In addition, the fact that leptin secretion is clearly up-regulated in obesity contributes to generate significant interest around this adipokine [57]. *In vitro* studies using recombinant leptin showed its ability to increase cancer cell proliferation via the activation of ERK1/2 and c-Jun NH2-terminal kinase (JNK) pathways [58]. Amemori *et al*. showed in a three dimensional collagen gel culture system, that adipocytes from wild-type mice increased the proliferation of colon cancer cells and that this effect was abolished in adipocytes from *ob/ob* mice deficient for leptin [47]. These results indicate that leptin enhances tumor cell growth *in vitro*. However, the *in vivo* role of leptin in tumorigenesis remains controversial. While *ob/ob* mice displayed decreased growth of colorectal and mammary tumors [59, 60], increased tumor growth was observed in prostate tumors [61]. Additionally, Aparicio *et al*. showed that leptin induced growth of colon cancer cells *in vitro* but not in nude mice and in *Apc^Min/+^* mice that were susceptible to spontaneous intestinal adenoma formation [62]. These apparent contradictory observations of tumor growth *in vivo* could be partly explained by the concurrent local and systemic effects of leptin on adjacent tumor cells and immune cells, respectively. Indeed, leptin was also shown to be important in development and cytotoxicity of immune cells [63, 64].

On the other hand, a number of human cancers were observed to express high levels of the adiponectin receptors (AdipoR1 and AdipoR2) [65–68], an adipokine whose secretion is down-regulated in obesity [69]. *In vitro* exposure of cancer cells to adiponectin inhibited proliferation and induced apoptosis in different cancer cell lines, such as breast, liver, colon, stomach and endometrium [65, 70–74]. *In vivo*, adiponectin reduced tumorigenesis of many cancer cells [72, 73, 75, 76], and adiponectin deficiency promoted tumor growth [77, 78]. The growth inhibition of adiponectin in cancer cells was shown to be mediated through activation of AMPK, inhibition of PI3K/Akt and ERK1/2 pathways, down-regulation of leptin-induced STAT3 phosphorylation, inhibition of NF-κB and Wnt/β-catenin pathways, and decrease of ROS production [79]. Thus, adiponectin mediates effects on tumor growth opposite to those of leptin.

Since both adipokines are secreted by adipocytes and present in the blood, the leptin:adiponectin ratio may be a major factor influencing tumor growth. It is worth noting that in obese patients, plasma concentration of leptin is increased, while that of adiponectin is decreased [57, 69]. Clinical studies have indicated a positive correlation between a high leptin:adiponectin ratio and an increased risk of postmenopausal breast [80, 81], colorectal [82] and endometrial cancers [83]. Interestingly, cancer-associated adipocytes displayed decreased expression of adiponectin [31], suggesting a hijacking of adipocytes by cancer cells to promote tumor growth.

### Insulin-like growth factor 1

High levels of circulating IGF-1 have been correlated with increased risk in many cancers, including premenopausal breast, prostate, lung and colorectal cancers [84]. Tumor cells were shown to express IGF-1R, and binding of IGF-1R to its ligand promotes cell growth and survival through activation of PI3K/Akt and MAPK pathways [84, 85]. Adipocytes were shown to secrete IGF-1, and obesity is associated with increased levels of IGF-1 [86]. Interestingly, inhibition of IGF-IR kinase activity prevented the growth-promoting effect of adipocytes on breast cancer cells [87].

### Hepatocyte growth factor

Hepatocyte growth factor (HGF) was shown to be secreted by human adipocytes and the production of HGF by adipocytes contributed to its elevated serum level in obesity [88]. In parallel, the HGF receptor (c-Met) was found to be expressed in breast and ovarian cancers [89, 90]. Interestingly, it was observed that in breast cancer samples, c-Met expression was increased at the adipocyte-tumor interface [91]. This expression pattern suggests a paracrine interaction between adipocytes and tumor cells. Rahimi *et al*. showed that HGF promoted proliferation of murine breast carcinoma SP1 cells [92]. Moreover, neutralization of HGF in the conditioned medium from murine 3T3-L1 adipocytes suppressed the adipocyte-induced proliferation of SP1 cells [92]. *In vivo*, overexpressing HGF in different tissues in mice resulted in tumorigenesis of those tissues [93]. Interestingly, a recent study by Sundaram in obese mice showed that weight loss reversed obesity-induced HGF/c-Met pathway and basal-like breast cancer progression [94].

### Estrogen

Estrogen receptors are expressed by cancer cells in breast and ovarian cancers [95]. Estrogen plays a crucial role in the promotion of growth in hormone-dependent cancers [96]. Although AT is a major source of estrogen, adipose stromal cells but not mature adipocytes were shown to express aromatase, the key enzyme in estrogen production [97, 98]. Interestingly, aromatase expression in stromal cells is induced by tumor cells via the secretion of PGE2 [99, 100]. Since CAAs were shown to acquire fibroblast-like characteristics [36], one might hypothesize that these adipocyte-derived fibroblasts could also produce estrogen and contribute to tumor growth. Importantly, in case of obesity, adipocytes can indirectly induce the expression of aromatase in breast cancer cells and AT via the recruitment of macrophages. Recent studies by *Arendt et al*. demonstrated that obese adipocytes secrete CCL2 and IL-1β which lead to the accumulation of macrophages surrounding dead adipocytes, forming crown-like structures [101]. Exposure of M1 macrophages to saturated fatty acids released as a result of obesity-associated lipolysis leads to the secretion of proinflammatory mediators, including PGE2, TNF-α, IL-1β, and IL-6. These molecules upregulate the expression of CYP19, the gene encoding estrogen synthase aromatase in adipose tissue and breast cancer epithelium, causing estrogen production and contributing to obesity-associated breast cancer [102].

### Other adipokines

IL-6 has been shown to promote tumor growth in different types of cancer [103]. Although IL-6 could be secreted by a various sources in the tumor microenvironment, including tumor cells, tumor-infiltrating macrophages, T cells, we and others have shown that IL-6 is highly secreted in CAA [28, 31]. Recently, Chen *et al*. demonstrated that marrow adipocytes in the vicinity of the tumor cells expressed high levels of IL-6, and blockade of IL-6 by neutralizing antibody blocked tumor growth of melanoma cells [49]. Intriguingly, the antitumor effect of anti-IL-6 antibody was only observed in high fat diet-induced obese mice but not in mice with normal diet [49]. This data underlines the importance of a pro-inflammatory microenvironment in obese conditions that could enhance tumor growth.

Resistin is another adipokine that has been linked to promoting tumor growth. Kim *et al*. showed that resistin induced prostate cancer cell proliferation through PI3K/Akt pathway [104]. In another study, Deshmukh *et al*. showed that resistin promotes growth and aggressiveness of breast cancer cells through STAT3 activation [105]. Adenylyl cyclase-associated protein 1 (CAP1) was identified as a functional receptor for human resistin to modulate inflammatory action in monocytes [106]. However, the expression of CAP1 or of other resistin receptors and their intracellular signaling pathways in different cancer cells remains to be determined. Circulating levels of resistin are increased in obesity [107], and epidemiological studies suggested a link between resistin levels and cancer risk [108].

Overall, above is a non-exhausted list of adipokines that could impact on tumor growth. It is worth noting that although the secretion of these adipokines is inherent property of adipocytes, it is strongly modulated along the acquisition of fibroblastic-like characteristics of CAA. While adipocyte terminal markers such as adiponectin, leptin and resistin are decreased in CAA, inflammatory cytokines such as IL-6 are increased. Further studies are required to better understand their individual contributions and their cooperation in tumor progression in the context of the tumor microenvironment, as well as the modulation of their corresponding receptors during tumor progression in different types of cancer.

### Adipocytes promote angiogenesis

Angiogenesis, the process of new blood vessel formation, plays a critical role in tumor expansion [109]. Blood vessels supply oxygen, nutrients, and growth factors from the plasma to tumor cells. In parallel, increase of adipose mass, in particular in obesity, is also associated with angiogenesis. This process is tightly regulated by tumor cells and stromal cells in the tumor microenvironment. It has been shown that adipocytes actively participate in angiogenic modulation through the secretion of adipokines [110, 111]. Classical angiogenic factors, such as vascular endothelial growth factor A (VEGFA), are produced by adipocytes in response to insulin [112]. Moreover, leptin could directly promote proliferation and angiogenic differentiation of endothelial cells which express leptin receptor [113]. Importantly, leptin was shown to upregulate VEGF in breast cancer via HIF-1 and NFκB; thus it could confer an additional advantage to tumors under hypoxic conditions [114]. Other adipokines, such as resistin, angiopoetin, and HGF also promote angiogenesis *in vitro* and *in vivo* [111, 115–118]. By contrast, the role of adiponectin in angiogenesis is debatable. While some studies showed that adiponectin may be pro-angiogenesis [77, 115, 116, 119, 120], other studies reported an inhibition of angiogenesis both *in vitro* and *in vivo* [121, 122]. Besides, certain adipocyte-derived lipids such as monobutyrin were also shown to induce angiogenesis [123]. Recently, Lim *et al*. showed that inoculation in WAT and BAT of different tumor types, including breast, melanoma and fibrosarcoma, resulted in marked increased of tumor growth rate relative to subcutaneous implementation [124]. Tumors implemented in WAT or BAT displayed augmented angiogenesis, blood perfursion and decreased hypoxia compared to subcutaneous tumors [124]. Interestingly, BAT is more efficient to induce tumor growth than WAT. The authors correlated this difference to the degree of pre-existing vascularization in those tissues. However, the impact of the adipocyte-cancer cell crosstalk on angiogenesis was not clear in this study and requires further investigations [124].

### Adipocytes provide energy to fuel tumor growth

The “Warburg effect”, in which oxidative phosphorylation is shifted to glycolysis to rapidly provide energy for tumor growth, even under normal oxygenic conditions, has been described in cancer cells several decades ago [125]. In parallel, cancer cells are capable of using alternative sources of energy, such as amino acids and lactate from the microenvironment [126, 127]. In 2009, Lisanti *et al*. proposed the “reverse Warburg effect”: cancer cells induce glycolysis in cancer-associated fibroblasts which in turn produce lactate and pyruvate for cancer cell metabolism and proliferation [128, 129]. This concept could be applied to other cells of the tumor stroma, notably adipocytes. These cells have been shown to release lactate through the monocarboxylate transporters, in particular under hypoxic conditions [130, 131]. However, several published studies demonstrated that the source of energy provided by adipocytes to cancer cells is lipids. As stated before, co-culture of adipocytes with breast cancer cells led to adipocyte delipidation [31]. In line with these data, we showed that CAAs located at the invasive front of breast cancer displayed smaller sizes and less lipid than adipocytes far from the tumors [31], an observation that has been largely confirmed by several groups in a wide range of solid tumors [25, 38, 132]. These phenotypic changes were initially associated with adipocyte “dedifferentiation” [31] but additional studies from our lab and others demonstrated that cancer cells also induce lipolysis in adipocytes [28, 133–135]. The free fatty acids (FFAs) liberated by adipocytes are then taken up by tumor cells and stored as lipid droplets to avoid lipotoxicity, both *in vitro* and *in vivo* [28, 48, 135]. Among FFAs, palmitic acid was shown to be the major FFA transferred from adipocytes to melanoma cells [48].

What is the fate of FFAs in cancer cells? In ovarian cancers, co-culture with adipocytes was associated with an up-regulation of the rate of fatty acid β-oxydation (FAO), a metabolic pathway that yields a large quantity of ATP [28]. At physiological levels, FAO is carried out in energy-demanding tissues (such as the heart and skeletal muscle) and recent works also brought to light a role for this metabolic pathway in cancer that seems to be highly responsive to environmental changes [136]. However, the involvement of this increased FAO in ATP production and growth-promoting effect of adipocytes has not been directly demonstrated in the ovarian cancer model [28]. In breast cancer, our most recent results demonstrated that increased FAO is dissociated from ATP production, this uncoupled FAO promoting an increase in invasion, but not proliferation, of cancer cells both *in vitro* and *in vivo* [135]. Therefore, although the lipid transfer between tumor-surrounding adipocytes and cancer cells appears to be a general phenomenon, the fate of these transferred FFAs might be dependent on the model studied. Similar lipid transfer between tumor cells and BM-Ad has been shown *in vitro* [39]. In opposition to the increase in FAO observed at primary sites, a recent study proposed that the crosstalk between prostate tumor cells and bone marrow adipocytes led to decreased mitochondrial oxidative phosphorylation in tumor cells associated to increased expression of glycolytic enzymes and increased lactate production via oxygen-independent mechanism of HIF-1α activation [133]. These differences suggest that the metabolic phenotype of tumor cells could also be dependent on the type of adipocytes surrounding the tumors.

What are the molecular actors that allow this transfer of FFAs between adipocytes and cancer cells? Fatty acid-binding protein 4 (FABP4) was identified as a key mediator in this process. In fact, FABP4 expression is increased in tumor cells cocultivated with adipocytes *in vitro* and in human tumors that invade AT [28]. Similar up-regulation of FABP4 was observed in tumor cells cocultivated with BM-Ad and in murine models of bone metastasis of prostate cancer [39]. Inhibition of FABP4 by small molecules reduced lipid accumulation in cancer cells in co-culture experiments with adipocytes, and *Fabp4*−*/*− mice presented significantly reduced ovarian tumor growth [28]. However, the factors that induce the first step of the crosstalk which is the initiation of lipolysis in adipocytes remain poorly described. Besides, it has been reported that exosomes derived from pancreatic cancer cells induce lipolysis in subcutaneous adipose tissue, exosomal adrenomedullin being a candidate mediator of this effect [137]. It remains to be determined whether this pathway might be operational in other tumor types. Besides, it is worth noting that induced lipolysis of adipose tissue contributes to cancer-associated cachexia [138], a syndrome characterized by the loss of adipose and muscle mass and frequently observed in untreated cancer patients [139]. Inflammatory cytokines made by host and/or tumor cells, such as TNF-α, IL-1, IL-6 and IFN-γ, have been reported to induce cancer cachexia in animal models [139]. In our hands, blocking TNFα or IL-6 was unable to prevent the delipidation of adipocytes induced by tumor cells in our co-culture system, suggesting that they are not involved and/or sufficient to explain this paracrine and “acute” delipidation [36]. Recently, Kir and *al*. identified parathyroid hormone-related protein (PTHrP), a tumor-derived small polypeptide, as an inducer of thermogenic gene expression and wasting in adipocytes [140, 141]. Neutralization of PTHrP by a specific antibody in tumor-bearing mice attenuated weight loss and mice lacking PTHrP receptor in adipose tissues are resistant to tumor-driven cachexia [140, 141]. Thus, PTHrP might represent an interesting candidate. Taken together, these data indicate that there is a metabolic crosstalk between adipocytes and cancer cells. Further studies are required to identify the key molecular actors in this process, the fate of transferred FFAs and its potential as a target in cancer therapy.

### Adipocytes promote tumor invasion and metastasis

Metastasis is a major cause of cancer-related death. Several clinical studies have shown a positive correlation between AT infiltration of different types of cancer and poor prognosis [86]. As we will see, during the invasion and metastatic processes, adipocytes can play important roles either by increasing cancer cell migration and invasion, remodeling of the extracellular matrix, or promoting tumor homing.

### Adipocytes increase cancer cell migration and invasion

Experiments showing the impact of adipocytes on cancer cell aggressiveness were mostly performed *in vitro* using co-culture system. We and others have shown that co-cultures of adipocytes with breast cancer cells increased cancer cell migration and invasion [31, 32, 36]. Moreover, co-cultured breast cancer cells displayed a downregulation of the epithelial marker E-cadherin and a reorganization of β-catenin without a simultaneous increase in mesenchymal markers [31]. These data suggest an incomplete induction of epithelial mesenchymal transition (EMT) by adipocytes. The invasion-promoting effect of adipocytes seems to not be dependent on cell-cell contact but mediated by soluble factors secreted by these cells. Interestingly, only the conditioned medium from CAAs, but not from “naive” adipocytes which had never been co-cultured with tumor cells, increased the invasive capacity of breast cancer cells [31]. Increased migratory and invasive abilities of tumor cells cocultivated with adipocytes have been observed with other models such as prostate cancer [46, 50]. Similar findings were obtained with prostate cancer cells cocultivated with BM-Ad [29]. This data strongly illustrates the cancer-adipocyte crosstalk and its importance in the modulation of tumor progression.

Several factors have been identified to be secreted by adipocytes and be involved in the invasion-promoting effect. In breast cancer, IL-6 was shown to be secreted by adipocytes, notably at high levels by CAAs [31]. Moreover, IL-6 promotes tumor migration and invasion, and its inhibition by blocking antibodies significantly reduces these effects [31]. Such an inflammatory state in the tumor-surrounding adipose tissue has also been described for prostate cancer. High levels of IL-6 were found in the peri-prostatic adipose tissue of tumor-bearing patients and the levels of IL-6 correlated with the aggressiveness of the tumors, highlighting the importance of this cytokine in the adipocyte/cancer cells crosstalk [27]. Another adipocyte-secreted factor, leptin, was shown to promote breast cancer cell migration and invasion via IL-18 expression and secretion [142]. Additionally, fatty acid transfer from marrow adipocytes could stimulate invasion of prostate cancer cells, which is decreased upon inhibition of FABP4 [143, 39]. Furthermore, we have recently uncovered that exosomes secreted by adipocytes promoted melanoma migration and invasion *in vitro* and *in vivo*, this effect being enhanced in case of obesity [144]. Exosomes are nanovesicles secreted by most cell types, which allow the transfer of lipids, proteins and nucleic acids between cells. A proteomic study further demonstrated that these vesicles carry proteins implicated in FAO, a feature highly specific to adipocyte exosomes. Transfer of these proteins from adipocytes to tumor cells is likely to promote the observed increase in tumor migration. In fact, in the presence of adipocyte-derived exosomes, FAO was increased in melanoma cells and pharmacological inhibition of this metabolic pathway completely abrogated the exosome-mediated increase in migration [144].

### Adipocytes remodel the extracellular matrix

Adipocytes secrete various constituents of the extracellular matrix, such as different types of collagen [145]. During tumorigenesis, cancer cells have been shown to upregulate the secretion of collagen VI by adipocytes [146]. Collagen VI was shown to promote tumor growth and survival via signaling through the NG2/chondroitin sulfate proteoglycan receptor expressed on tumor cells [147]. In the case of induced expression of the mouse mammary tumor virus/polyoma virus middle T oncogene, mice lacking collagen VI exhibited reduced rates of hyperplasia and primary tumor growth. Particularly, adipocytes from collagen-deficient mice were less potent in stimulating tumor growth [147]. Furthermore, endotrophin, a cleaved fragment of the collagen VI α3 chain, promoted the EMT process and metastatic spread of mammary epithelial cancer cells [148].

Adipocytes also secrete a number of matrix metalloproteinases (MMP) which allow the remodeling of the extracellular matrix [149]. Many MMPs have been shown to be involved in the promotion of tumor invasion [150]. Importantly, cancer cells were shown to induce the expression of MMP-11 in adipocytes as tumor invaded the surrounding adipose tissue [34]. Although the substrates of MMP-11 remain unknown [151], some studies suggested that MMP-11 can cleave collagen VI [152]. *In vivo*, MMP-11 was shown to promote cancer progression by remodeling the extracellular matrix and downregulation of MMP-11 by siRNA attenuated cancer metastasis [153–155]. Additionally, high levels of MMP-11 expression were correlated with increased invasion and bad prognosis in breast carcinoma [156], pancreatic cancer [157] and colon cancer [158]. In parallel, MMP-11 can directly act on adipocytes and negatively regulate adipogenesis by inhibiting adipocyte differentiation and by enhancing dedifferentiation, leading to accumulation of fibroblast-like cells in the tumor microenvironment [152]. Furthermore, adipokines such as leptin or HGF can induce the secretion of various MMPs by cancer cells, thus indirectly promoting tumor invasion [159–161].

### Adipocytes promote tumor homing and seeding at distant organs

Adipose tissues are preferential metastatic sites of several cancers. Nieman *et al*. showed that omentum adipocytes favored tumor homing via secretion of cytokines and chemokines [28]. The most abundantly adipocyte-secreted cytokines included IL-6, IL-8, MCP-1, and tissue inhibitor of metalloproteinase-1 (TIMP-1). Antibody-mediated inhibition of these factors reduced ovarian cancer cell homing toward adipocytes *in vitro* [28]. Neutralization of IL-6R and particularly IL-8R (CXCR1) reduced *in vivo* homing of ovarian cancer cells to the mouse omentum [28]. Notably, the expression of IL-8R was strongly upregulated in ovarian cancer cells in co-culture with adipocytes. In another study, Pramanik *et al*. showed that acute lymphoblastic leukemia (ALL) cells migrated into adipose tissues *in vivo* and exhibited chemotaxis towards adipocytes *in vitro* [162]. CXCL12/SDF-1 was identified as an adipocyte-derived chemoattractant responsible for leukemia cell migration. Inhibition of the SDF-1 receptor, CXCR4, in ALL cells abrogated their migration toward adipocytes. In prostate cancer (PCa), we uncovered that periprostatic adipose tissue (PPAT) was able to support the directed migration of tumor cells, therefore favoring the dissemination of the cancer outside of the prostate gland [163]. The secretion, by mature adipocytes of the periprostatic fat, of the chemokine MCP-3/CCL7 supports this process and within the context of obesity this secretion is increased. The receptor for this chemokine, CCR3, has been identified in PCa cells. Blocking the CCL7/CCR3 axis inhibited PCa local invasion *in vitro* and *in vivo* with a striking effect in obese animals. More importantly, expression of this receptor was associated with aggressiveness in PCa patients and was correlated with extra-prostatic dissemination and surgical treatment failure [163]. Finally, it has been suggested using human bone tissue fragments that breast cancer cells exhibit *in vitro* directed migration towards BM-Ad that correlated with leptin and IL1Δ levels of expression [164], a process that needs to be confirmed *in vivo*.

**Figure 2:**
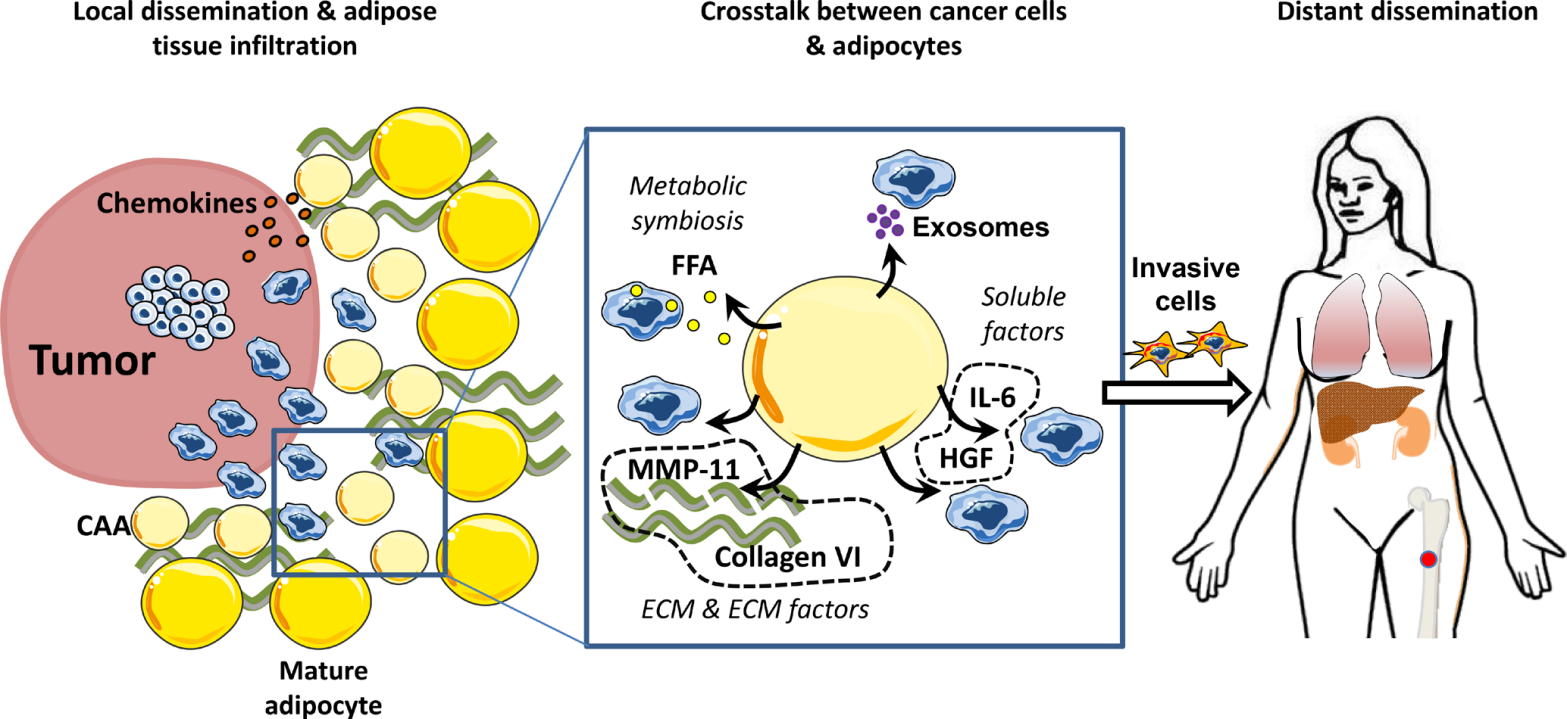
Adipocytes promote tumor invasion and metastasis Secretion of cytokines and chemokines by mature adipocytes favor the homing of tumor cells to surrounding adipose tissue. Once the adipose tissue is invaded, a crosstalk is established between cancer cells and mature adipocytes that undergo phenotypical changes towards a Cancer Associated Adipocytes (CAAs) phenotype. Their ability to secrete soluble factors, exosomes and extra-cellular matrix components stimulate invasive properties of tumor cells. These invasive cells can enter the blood stream and colonize distant organs including bone which is also an adipocyte-rich organ.

Altogether, these data illustrate that mature adipocytes can act at each key step of the metastatic process, the main findings being summarized in Figure 2. These effects are mediated through the release of soluble factors (including pro-inflammatory cytokines and chemokines), ECM components as well as through the active release of exosomes. The relative contribution of these different mechanisms remains unknown. Increase in invasive capacities is a very important aspect of the adipocyte/cancer crosstalk. These results should be further considered in light of the clinical studies that show an increase in local and distant dissemination of both prostate and breast cancers in obese patients [165].

### Adipocytes increase cancer cell survival and resistance to therapies

Several studies have shown that stromal cells such as fibroblasts could promote survival and drug resistance in cancer [166]. However, little is known about the impact of adipocytes in the efficiency of anti-cancer therapies. The first evidence of the protective role of adipocytes on cancer cells came from the observation by Iyengar *et al*., who showed that 3T3-L1 adipocytes supported survival of transformed ductal epithelial cells *in vitro* under limiting serum conditions [146]. Other epidemiological and experimental studies, including ours, have demonstrated the involvement of adipocytes in the evasion of cell death and promotion of cancer resistance to therapies.

### Epidemiological evidence linking obesity and tumor resistance to therapies

As stated before, obesity has been clearly associated with higher mortality in obese cancer patients compared to lean cancer patients [167]. This could be partly due to the presence of more advanced diseases in obese patients at the time of diagnosis [17]. Moreover, the under-dosing of therapy regimens by clinicians due to the fear of toxicity could also contribute to the decrease in treatment efficacy [168]. Accordingly, recent recommendations highlighted that full weight–based chemotherapy doses need be used in the treatment of obese patients with cancer, especially when the goal of treatment is cure [168]. In addition, it has also been proposed that obesity can alter the pharmacokinetics of drugs used in chemotherapy [142, 143]. However, independently of these parameters, clinical data have shown that obesity could have an independent effect in cancer resistance to therapies [165].

Concerning the impact of AT on sensitivity to radiotherapy, the results remain controversial since some studies have shown an adverse effect of obesity on clinical outcome of prostate cancer patients [171–173], while other reports have indicated no difference in efficacy between obese and lean patients in prostate and esophageal cancers [174–176]. By contrast, the link between obesity and resistance seems to be more firmly established for chemotherapy and endocrine therapy. In a study by Ewertz and colleagues on 53,816 women with early-stage breast cancer treated with different regimens of chemotherapy or endocrine therapy (tamoxifen or aromatase inhibitors), the authors observed an increased risk of developing distant metastases in obese patients compared to lean patients [177]. In addition, both chemotherapy and endocrine therapy were found to be less effective in obese patients after 10 or more years of follow-up [177]. This finding of a negative impact of obesity in breast cancer was supported by another retrospective study by Jiralerspong on 6,342 patients with breast cancer [178]. In regards to targeted therapy, the impact of obesity on outcome has been investigated in only a few studies. In colorectal cancer, three retrospective trials have been conducted with conflicting conclusions. While Simkens *et al*. found that an increase in BMI was associated with a better overall survival in patients treated with chemotherapy, but not in patients treated with chemotherapy and bevacizumab, an anti-VEGF antibody [179]. Conversely, Guiu *et al*. showed that high BMI values and large visceral fat content were associated with poorer response, shorter time-to-progression and shorter overall survival in patients treated with bevacizumab and chemotherapy, but there was no significant association in patients treated with chemotherapy alone [180]. The negative impact of adiposity was supported by a study by Patel *et al*., in which the authors concluded that overweight BMI represented an independent, poor prognostic indicator for survival in patients undergoing chemotherapy with and without targeted therapy [181]. Besides, in breast cancer, Crozier *et al*. reported that adjuvant trastuzumab, an anti-HER2 antibody, improved the clinical outcome in a cohort of 3,017 patients regardless of their BMI [182].

Overall, further epidemiological studies are required to better understand the impact of high body adiposity on cancer survival, whether this was the case before or after diagnosis. Interestingly, several studies suggested that weight gain after diagnosis appeared to be associated with greater disease mortality [165]. However, whether weight loss after diagnosis may improve prognosis remains unclear and requires further investigations [165].

### Adipocytes protect cancer cells via ECM

One of the aspects of the environmental protection of cancer cells is the increase of cell adhesion to the extracellular matrix [183, 184]. These interactions lead to the reorganization of cell cytoskeleton and activation of multiple transduction pathways, resulting in increased cell survival and resistance to chemotherapeutic agents. As previously mentioned, adipocytes are an abundant source of extracellular matrix components. Particularly, adipocytes which are in close contact with cancer cells secrete high levels of collagen VI [147]. *In vitro* exposure of tumor cells to collagen VI conferred resistance of ovarian cancer cells to cisplatin [185], possibly via the upregulation of metallothioneins which are known to play critical role in cisplatin resistance [186, 187]. Park *et al*. further showed that endotrophin, a cleavage product of collagen VI alpha 3 chain, enhanced EMT and caused resistance to cisplatin [188]. Interestingly, the same group showed that adipocyte-derived endotrophin could also induce adipose tissue fibrosis and inflammation [189], indicating mutual effects between tumor cells and CAAs.

### Adipocytes protect cancer cells via secretion of adipokines, metabolites or exosomes

As previously discussed, adipocytes are source of various paracrine factors and many of them have been shown to be responsible for cancer resistance to therapies.

The main mechanisms involved in adipocytes-induced resistance have been the modulation of cell death pathways. Behan *et al*. showed that adipocytes protected acute lymphoblastic leukemia cells from cytotoxic agents, including vincristine, dexamethasone, daunorubicin, and nilotinib *in vitro* [190]. This protection was independent of cell-cell contact and was associated with increased expression of pro-survival signals Bcl-2 and Pim-2 in cancer cells [190]. In another study, Chi *et al*. showed that adipocyte-secreted leptin contributed to resistance of melanoma cells to various therapeutic agents, including cisplatin, docetaxel, and the histone deacetylase inhibitor SAHA [191]. This was associated with increased activation of survival pathways PI3K/Akt and MEK/ERK. Enhancement of prosurvival pathways by leptin has also been shown to counteract the cytotoxic effect of 5-fluorouracil, a common therapeutic agent for colon cancer [192]. In the same line of findings, we previously reported that mature adipocytes could protect breast cancer cells from ionizing radiation-inducing post-mitotic cell death. This effect was due to the secretion of IL-6 by tumor cells, secretion that was upregulated in the presence of adipocytes [193]. In another study, we showed that adipocytes could protect HER2-positive breast cancer cells from trastuzumab-mediated cellular cytotoxicity *in vitro* and from antitumor effect of trastuzumab *in vivo* [194]. Interestingly, this protective effect was enhanced under hypoxic conditions [194], underlying the importance of adipocytes in the tumor microenvironment and/or obesity. Adipocyte-secreted factors rapidly activated Akt survival pathway in cancer cells and upregulated the expression of several genes involved in cell survival [194]. More importantly, the protection of tumor cells by adipocytes seems not to be limited to monoclonal antibodies such as trastuzumab or the drug-antibody conjugate T-DM1, but also to kinase inhibitors (data not shown). Furthermore, in a recent study, it has been demonstrated that adipocytes could protect cancer cells by downregulating APAF1, a key protein involved in the formation of apoptosomes [195]. APAF1 was shown to be a direct target of miR21, which is abundantly present in exosomes isolated from CAAs and CAFs [195]. The authors showed that miR21 was transferred by exosomes from CAAs or CAFs to cancer cells, where it suppressed ovarian cancer apoptosis and conferred chemoresistance to paclitaxel by binding to its direct target, APAF1 [195].

Resistance to drugs induced by adipocytes is not limited to modulation of apoptosis. Pramanik *et al*. also demonstrated that both subcutaneous and visceral fat pads from obese and control mice protected acute lymphoblastic leukemia (ALL) cells from chemotherapy [162]. This protection was mediated via ALL-induced oxidative stress response in adipocytes and secretion of soluble factors by adipocytes [196]. However, the adipocyte-derived factors mediating resistance to chemotherapeutic agents were not clearly identified in these studies. Resistance to vincristine was also observed *in vivo* [190] and additional studies demonstrated that obesity altered vincristine pharmacokinetics in blood and tissues of mice, highlighting again that resistance to drugs induced by obesity is probably multifactorial [169]. Besides, a recent study by Liu *et al*. demonstrated that bone marrow adipocytes protected myeloma cells against chemotherapy through autophagy activation [197]. Leptin and adipsin were identified to be secreted by adipocytes and to be responsible for this mechanism of resistance [197].

In parallel, adipocytes could directly counteract the effects of chemotherapeutic agents by acting as source of metabolites, by modulating drug transport or intra-cellular metabolism. L-asparaginase is a first-line therapy for acute lymphoblastic leukemia (ALL) that breaks down asparagine and glutamine, two amino acids important in the metabolism of ALL. Ehsanipour *et al*. showed that adipocytes caused leukemic cell resistance to L-asparaginase via the release of glutamine [198]. Interestingly, these protective effects were observed with bone marrow-derived adipocytes in obesity, notably after the induction of chemotherapy. Another study showed that adipocyte-conditioned medium decreased tumor cell response to gemcitabine *in vitro*. This increase in resistance seems to be due to modification of the expressions of genes involved in gemcitabine transport and metabolism in tumors [199].

Altogether, these data indicate that adipocytes could enhance cancer resistance to different types of therapies, through a variety of mechanisms (Table 1). They also suggest that the local microenvironment as well as metabolic factors need to be taken into account not only in assessing cancer risk, but also in the design of clinical trials in oncology.

**Table 1:** Adipocytes increase cancer resistance to therapies

Mechanism	Tumor model	Adipocyte model	Therapeutic agents	Observation	Reference
Enhanced EMT process	Mammary tumors	Murine adipose tissue	Cisplatin	COL6−/− mice or thiazolidinediones or endotrophin neutralizing antibody sensitize tumors to cisplatin treatment *in vivo*	[188]
Upregulation of survival gene expression and pro-survival pathways	Human and murine leukemia cell lines	3T3-L1 murine cell line, OP-9 murine bone marrow-derived cell line	Vincristine, dexamethasone, daunorubicin and nilotinib	Obesity impairs the effect of vincristine in mice.	[190]
		Diet-induced obese mice		Co-culture with adipocytes decreases chemotherapy-induced cytotoxicity on leukemia cells *in vitro*	
	Human breast cancer cell lines	3T3F442A murine cell line	Ionizing radiation	Adipocytes lower ionizing radiation-induced cell death *in vitro*	[193]
		hMAD human cell line	Trastuzumab	Adipocyte-conditioned medium reduces trastuzumab-induced antibody-dependent cellular cytotoxicity on tumor cells *in vitro*	[194]
		Human adipose tissue		Adipose tissue reduces trastuzumab-induced cytotoxicity on tumor *in vivo*	
	Melanoma cell line	Human adipocytes	Cisplatin, docetaxel, and the histone deacetylase inhibitor SAHA	Adipocyte-conditioned media reduces sensitivity to treatment-induced apoptosis of melanoma cells *in vitro*	[191]
	Human colorectal tumor stem cells		5-fluorouracil	Leptin counteracts cytotoxic effects of 5-fluorouracil *in vitro*	[192]
	Human ovarian cancer cell lines	Adipocytes isolated from omental tissues of patients	Paclitaxel	Downregulation of APAF1 by adipocyte-derived exosomal microRNA21 enhances chemoresistance *in vitro* and *in vivo*	[195]
Increase of oxidative stress response	Human and murine leukemia cell lines	Subcutaneous and visceral fat pads from obese and control mice	Daunorubicin	Co-culture with adipocytes decreases chemotherapy-induced cytotoxicity on leukemia cells	[162, 196]
		Murine 3T3L1 and human Chub-S7 adipocyte cell lines			
Autophagy activation	Multiple myeloma (MM) human cell lines	Bone marrow isolated adipocytes from human, subcutaneous fat and human pre-adipocyte cell line PCS-210-010	Melphalan, bortezomib, dexamethasone, and doxorubicin	Adipocyte-conditioned media inhibit chemotherapy-induced apoptosis of MM cells *in vitro* and *in vivo*	[197]
	Bone marrow-isolated MM cells from patients				
Adipocyte-secreted glutamine	Murine and human leukemia cell lines	3T3-L1 murine cell line, OP-9 murine bone marrow-derived cell line	L-asparaginase	Obesity impairs L-asparaginase efficacy in mice	[198]
	Human primary leukemia cells	Diet-induced obese mice		Adipocytes inhibit the treatment-induced tumor cell apoptosis *in vitro*	
Dysregulation of genes involved in gemcitabine transport and metabolism	Human and murine mammary cancer cell lines	3T3-L1 murine cell line	Gemcitabine	Adipocyte-conditioned medium decreases tumor cell response to gemcitabine *in vitro*	[199]
		Diet-induced obese mice		Obesity induces resistance to gemcitabine *in vivo*	

## CONCLUSIONS

Over the two past decades, with the increasing rates of obesity and metabolic disorders, as well as their link to cancer, adipocytes have increasingly received attention of researchers and clinicians. Growing evidence has transformed adipocytes from a passive, neutral cell type into an active actor playing important roles in both metabolic homeostasis and shaping of the microenvironment. Given the large volume of adipose cells and their proximity with cancer cells, adipocytes need to be considered as a major component in the microenvironment in many solid cancers, including breast, colon, prostate, ovarian cancers, as well as in hematological malignancies. It is important to underline the crosstalk between adipocytes and cancer cells, which leads to a profound modification of the adipocyte phenotype as well as the adipocyte secretome. These CAAs could strongly support cancer progression through different axes: 1) they constitute a source of metabolites and adipokines to fuel tumor growth; 2) they promote invasive properties of tumor cells both at the primary tumor site and distant metastases; 3) they protect cancer cells against various therapies. Since obesity has become a global epidemic and it is commonly associated with poor prognosis in many cancers, it is of fundamental and clinical interest to further study the relationship between adipose tissue and cancer cells. Better characterization of CAAs and the key molecular events in the adipocyte-cancer cell crosstalk will provide insights into tumor biology and suggest efficient therapeutic opportunities.
